# Improving Triterpenoid Extraction Efficiency from *Inonotus hispidus* Using Macroporous Adsorption Resin: An Aim for Functional Food Development

**DOI:** 10.3390/foods14061069

**Published:** 2025-03-20

**Authors:** Shuhan Dong, Shuliang Liu, Shilong Wang, Zhengliang Qi, Qianqian Zhuang, Xinli Liu

**Affiliations:** 1 State Key Laboratory of Bio-Based Material and Green Papermaking (LBMP), Qilu University of Technology (Shandong Academy of Sciences), Jinan 250353, China; 19861407319@163.com (S.D.); 13964311587@163.com (S.L.); 18266613391@163.com (S.W.); zqq0608@126.com (Q.Z.); liuxl@qlu.edu.cn (X.L.); 2 Key Laboratory of Shandong Microbial Engineering, College of Bioengineering, Qilu University of Technology (Shandong Academy of Sciences), Jinan 250353, China

**Keywords:** *I. hispidus*, high triterpenoid yield, macroporous adsorption resin, triterpenoid extraction and enrichment

## Abstract

The triterpenoids from *Inonotus hispidus* exhibit several valuable bioactivities, including antioxidant, anti-inflammatory, and anticancer properties, making them highly sought-after for applications in functional foods. To obtain more *I. hispidus* triterpenoids with higher content for subsequent application in functional food, this study firstly screened a strain with a high triterpenoid yield for the cultivation of fruiting bodies. Afterwards, the static adsorption and desorption capacities of seven macroporous adsorption resins (MARs) for fruiting body triterpenoids were evaluated via static tests, and MAR HPD-600 showed the best performance. The static adsorption kinetics and isotherms of triterpenoids were analyzed further using MAR HPD-600, revealing that the adsorption process followed pseudo-second-order kinetics and the Freundlich model, indicating a spontaneous exothermic reaction. The dynamic adsorption–desorption parameters of MAR HPD-600 were subsequently evaluated to establish the optimal enrichment process. With the optimal strategy, the content of triterpenoids in the desorption solution of MAR HPD-600 increased from 26.72 to 129.28 mg/g, with a high yield of 75.48%. Conclusively, the application of MAR presents an efficient and cost-effective technique for extracting *I. hispidus* triterpenoids, making it well-suited for functional food production requirements.

## 1. Introduction

*Inonotus hispidus* is a valuable edible and medicinal fungus, which is widely found in China, in provinces such as Shandong, Xinjiang and Hebei. It often grows on mulberry trees and has a yellow color, and thus is also known as “Sanghuang” [1]. Studies have revealed that *I*. *hispidus* is rich in several bioactive compounds, such as triterpenoids, polyphenols and polysaccharides [2]. Modern pharmacological studies have shown that *I*. *hispidus* has various biological properties, including antitumor [3], antioxidant [4], antiviral [5] and immune-enhancing [6] effects, which underscore its potential development value across the fields of functional food and medicine. For example, Sanghuang wine can induce tumor cell apoptosis through the mitochondrial apoptotic pathway and the death receptor pathway [7].

Triterpenoids are a large category of compounds with important bioactivity, commonly found in fungi, such as *Ganoderma lucidum* [8] and *Physisporinus vitreus* [9]. Triterpenoids possess a variety of important biological properties, such as being hypoglycemic [10], anticancer [11], anti-inflammatory [12] and neuroprotective [13]. To date, more than 15 triterpenoids have been isolated from *I*. *hispidus* [14]. Ren et al. demonstrated that inotolactone B effectively activated melanogenesis and tyrosinase activity in B16 melanoma cells, outperforming 8-MOP, indicating its potential as an anti-vitiligo agent [15]. Studies also revealed that the activity of triterpenoids is positively correlated with their dosage within a certain concentration range [16]. In summary, the selection of easy-to-operate and efficient extraction and enrichment methods is crucial for enhancing the utilization value of triterpenoids from *I. hispidus* and advancing their industrial application in the field of functional food.

The common methods for extracting and purifying terpenoids mainly include silica gel column chromatography [17], microwave-assisted [18] and supercritical fluid extraction [19]. Triterpenoids generally exhibit low polarity, and their structures are relatively complex with large molecular sizes [20]. During silica gel column chromatography extraction, they may undergo reorganization or degradation. This consumes a large quantity of organic solvents that may pollute the environment. The microwave-assisted method is unfavorable for the extraction of heat-sensitive triterpenoids [21]. Supercritical fluid extraction is costly and unfavorable for the extraction of polar compounds. Furthermore, supercritical carbon dioxide (CO₂) is commonly used for extracting low-polarity compounds, but its interaction with the complex cyclic structures and non-polar regions of triterpenoids may be suboptimal. In conclusion, the corresponding disadvantages make them less suitable for the industrial scale extraction of triterpenoids from *I*. *hispidus*. Therefore, it is necessary to find a cost-saving, simple, and green method. Using an MAR is a widely used method for extracting and purifying target compounds from plants and fungi, and is characterized by effective separation, reusability and cost-efficiency, making it better suited for industrial applications [22]. MARs have been applied to separate, extract, and enrich terpenoid components. Researchers have utilized MARs to effectively obtain diterpenoids from *Wikstroemia chamaedaphne* [23] and *Symphytum officinale* L. root [24]. Nevertheless, the study of MARs for the enrichment of *I*. *hispidus* triterpenoids has not been reported.

In this study, an MAR was used to extract and purify triterpenoids from *I*. *hispidus*. The adsorption and desorption properties of various MARs were assessed to identify the most effective one for triterpenoid adsorption, and the underlying adsorption mechanism was investigated. Additionally, the dynamic adsorption and desorption processes of MARs were optimized. Finally, an efficient method for extracting and purifying triterpenoids from *I*. *hispidus* was developed, thus providing valuable information for industrial application.

## 2. Materials and Methods

### 2.1. Strains and Materials

All *I. hispidus* strains used in this study were preserved in our laboratory. 

Potato dextrose water medium (PDB, Hope Bio-Technology Co., Ltd., Qingdao, China), MARs AB-8, D101, HPD-600, S-8, X-5, NKA-2 and NKA-9 (Baoen Resin Technology Co., Ltd., Cangzhou, China), oleanolic acid standard (CATO Research Chemicals Inc., Guangzhou, China), vanillin (Kemiou Chemical Reagent Co., Ltd., Tianjin, China), glacial acetic acid (Sinopharm Chemical Reagent Co., Ltd., Shanghai, China), perchloric acid (Sinopharm Chemical Reagent Co., Ltd., Shanghai, China), ultraviolet spectrophotometer (Metash Instruments Co., Ltd., Shanghai, China), ultrasonic cleaner (Ultrasonic Instruments Co., Ltd., Kunshan, China), L100–1S-2 peristaltic pumps (Lange Constant Flow Pump Co., Ltd., Baoding, China), chromatography column (Huxi Analysis Instrument Factory Co., Ltd., Shanghai, China), acetonitrile( Macklin Biochemical Technology Co., Ltd., Shanghai, China), formic acid (Macklin Biochemical Technology Co., Ltd., Shanghai, China), methanol (Macklin Biochemical Technology Co., Ltd., Shanghai, China).

### 2.2. Culture Media of Fruiting Bodies

The solid cultivation medium (*w*/*w*) consisted of 70% mulberry branch sawdust, 20% wheat bran, 8% corn flour, 1% calcium carbonate and 1% glucose, with a medium-to-liquid ratio of 1:1.2. Firstly, all the components were thoroughly mixed. Subsequently, the well-blended mixture was packaged into polypropylene plastic bags, with each bag containing approximately 500 g of dry material. These bags were then subjected to sterilization at 121 °C under high pressure for 30 min. After the sterilization process, they were allowed to cool down and were stored for future use.

### 2.3. Strain Cultivation

The strain cultivation was conducted following the method of Cheng et al. (2020) with slight modifications [25]. For the submerged cultivation of *I. hispidus* strains, mycelial lawns with consistent growth were initially selected. Then, three 7-mm-diameter pieces of fungal mycelium were excised from the colony edges and inoculated into 250-mL conical flasks containing 150 mL of PDB medium. The flasks were immediately incubated in a shaker at 30 °C with a rotation speed of 1.79× *g* for 11 days. Afterward, the mycelial mats were collected, rinsed 4–5 times with deionized water, and then placed in an oven at 60 °C until they reached a constant weight. Finally, the dried mycelium was passed through a 60-mesh sieve for further analysis.

### 2.4. Cultivation of Fruiting Bodies

Mycelial blocks with a diameter of 1 cm were selected and transferred into sterilized solid cultivation bags. Each bag was inoculated with 3–5 mycelial blocks, ensuring an even distribution across the surface of the medium. After inoculation, the bag openings were immediately sealed with an elastic band to prevent contamination by foreign microorganisms. The inoculated bags were then placed in an incubation room and cultivated at 30 °C in the dark for 25 days. Once the mycelium fully colonized the cultivation bags, the bags were transferred to the fruiting room for fruiting body induction. At this stage, the temperature in the incubation room was reduced to 24 °C, and the day-night temperature differential was increased to 5–8 °C. The relative humidity was maintained at 85%, and diffuse light with an intensity of 300× was provided for 10–12 h per day. Simultaneously, the concentration of carbon dioxide in the air was reduced. Under these induction conditions, fruiting bodies of SH-18 were obtained after 30 days of cultivation.

### 2.5. Pretreatment of MAR

The physical parameters of the seven MARs were summarized in Table 1. The MARs were pretreated according to the method described by Wu et al. [26]. The surfaces of the MARs were first cleaned with distilled water and then soaked in 95% (*v*/*v*) ethanol for 24 h to remove preservatives and surface impurities. Afterward, the MARs were thoroughly washed with distilled water until no alcohol odor was detectable. They were then soaked in 6% (*v*/*v*) HCl for 3 h, and the pH was subsequently adjusted to neutral with distilled water. Next, the MARs were immersed in 6% (*v*/*v*) NaOH for 3 h and rinsed with distilled water until a neutral pH was achieved. Finally, the treated MARs were soaked in distilled water and stored for later use.

### 2.6. Preparation of the Crude Extract of Triterpenoids

For extraction, the mycelium powder was mixed with 70% (*v*/*v*) ethanol at a 1:30 ratio. The crude extract was obtained through ultrasonic extraction at 50 °C, 220 V, and 50 Hz for 40 min. The mixture was then centrifuged at 8319× *g* for 10 min at 20 °C, and the supernatant was collected.

The fruiting bodies of SH-18 were cut into pieces and dried in an oven at 60 °C until their weight stabilized. They were then ground into powder using a high-speed pulverizer, sieved through a 60-mesh screen, and stored at 4 °C. Triterpenoids in the fruiting bodies were extracted following the same procedure used for the mycelium. These extracts were concentrated using rotary evaporation at 42 °C and subsequently freeze-dried into a powder. The resulting powder was stored in a freezer at −80 °C for use in subsequent experiments.

### 2.7. Determination of Total Triterpenoid Content

The triterpenoid content was determined according to NY/T 3676-2020 (Ministry of Agriculture and Rural Affairs of the People’s Republic of China, 2020) [27]. First, a standard curve for triterpenoids was established. The oleanolic acid standard solution was diluted to various concentrations and added to a 10-mL glass colorimeter. The solvent was then evaporated using a water bath nitrogen evaporator. Next, 0.1 mL of a 5% vanillin–glacial acetic acid solution and 0.8 mL of perchloric acid were added and mixed thoroughly, and the mixture was incubated in a water bath at 60 °C for 20 min to allow the color to develop. Subsequently, the tube was placed in an ice-water bath to cool for 3–5 min. Then, 5 mL glacial acetic acid was added, and the mixture was left at room temperature for 10 min. The absorbance was measured at 550 nm. A standard curve was constructed with the mass of the oleanolic acid standard on the horizontal axis and absorbance on the vertical axis. For sample analysis, a suitable volume of the sample was pipetted into a 10-mL glass colorimeter tube and processed using the same procedure. A blank control was also prepared simultaneously. The absorbance of the sample was measured, and the triterpenoid content was calculated by comparing the absorbance with the standard curve.

### 2.8. Static Adsorption–Desorption of MAR

#### 2.8.1. Screening of MAR

The optimal MAR was selected for subsequent experiments by comparing the adsorption and desorption rates of different MARs on the target substances. A total of 1 g of each of the seven pretreated MARs was placed in 50-mL conical flasks, and 20 mL of a sample solution containing 0.672 mg/mL of triterpenoids was added to each flask, with three replicates for each MAR. The flasks were then incubated in a shaker at 30 °C and 200 rpm for 24 h. The adsorption capacity and rate of each MAR for triterpenoids were determined by measuring the triterpenoid content in the adsorbed solution. Subsequently, the desorption rate was assessed. The sample liquid in each flask was filtered out, and the MAR was washed with distilled water until no residual sample liquid remained on the surface, after which it was wiped dry. Each flask received 30 mL of 95% (*v*/*v*) ethanol as the eluent, and the flasks were placed in a shaker at 30 °C and 200 rpm for 12 h, with three replicates for each type of MAR. The adsorption rate (*Q*) of each resin and equilibrium adsorption capacity (*Q_e_*) were calculated according to Equation (1) and (2), respectively. The desorption rate (*D*) (%) was calculated according to Equation (3).

(1) $$Q=\frac{C_{0}-C_{e}}{C_{0}}*100\%$$

(2) $$Q_{e}=\frac{\left(C_{0}-C_{e}\right)*V_{i}}{W}$$

(3) $$D=\frac{C_{d}V_{d}}{\left(C_{0}-C_{e}\right)*V_{i}}$$

Here, *C*_0_ and *C_e_* represent the initial and equilibrium concentrations of triterpenoids from *I*. *hispidus* (mg/mL), respectively. *V_i_* is the volume of the sample solution (mL), and *W* is the wet weight of the MAR (g); *C_d_* is the triterpenoid concentration in the eluent (mg/mL), and *V_d_* is the eluent volume (mL).

#### 2.8.2. Adsorption Kinetics

We precisely weighed 1 g of the MAR selected in Section 2.8.1 and transferred it into a 50-mL conical flask. We added 20 mL of the sample solution to each flask, and incubated it at 30 °C with constant shaking at 200 rpm for 24 h. Samples were collected from the flasks at regular intervals (every hour), and the triterpenoid content was quantitatively analyzed. All experiments were performed in triplicate to ensure reproducibility. To further elucidate the adsorption behavior of triterpenoids on the MAR, the adsorption kinetics were evaluated using pseudo-first-order Equation (5), pseudo-second-order Equation (6), and intraparticle diffusion kinetic Equation (7) models. The mathematical equations for these kinetic models are as follows:

(4) $$Q_{t}=\frac{(C_{0}-C_{t})V_{0}}{m}$$

(5) $$\ln\left(Q_{e}-Q_{t}\right)=\ln Q_{e}-\frac{k_{1}t}{2.303}$$

(6) $$\frac{t}{Q_{t}}=\frac{t}{k_{2}Q_{e}^{2}}+\frac{t}{Q_{e}}$$

(7) $$Q_{t}=k_{i}t^{0.5}+C_{i}$$

where *Q_t_* is the MAR adsorption capacity (mg/g) at time *t* (h). *C_t_* is the triterpenoid content (mg/mL) at time *t*. *k*_1_ and *k*_2_ [g/(mg·min)] are the rate constants for the pseudo-first-order and pseudo-second-order models, respectively. *k_i_* (mg/g/min) is the empirical constant for the intraparticle diffusion model.

#### 2.8.3. Adsorption Isotherm Evaluation

The adsorption isotherm describes the relationship between the concentration of the target compound and the adsorbent under specific temperature conditions. In this study, 1 g of the selected MAR was accurately weighed and transferred into five 50-mL conical flasks, each containing sample solutions with varying triterpenoid concentrations, forming an experimental group. These flasks were incubated at three controlled temperatures (25 °C, 30 °C, and 35 °C) with constant agitation at 200 rpm for 6 h. The adsorption behavior at each temperature was evaluated by determining the adsorption capacity and equilibrium concentration. All experiments were conducted in triplicate to ensure reliability. To model the adsorption process, the Langmuir, Freundlich, and Temkin isotherm models were applied to establish the corresponding isothermal equations:

Langmuir equation

(8) $$\frac{C_{e}}{Q_{e}}=\frac{1}{K_{L}Q_{m}}+\frac{C_{e}}{Q_{m}}$$

Freundlich equation

(9) $$\ln Q_{e}=\frac{1}{n}\;\ln C_{e}+\ln K_{F}$$

Temkin equation

(10) $$Q_{e}=B_{T}\ln C_{e}+B_{T}\ln K_{T}$$

where *K_L_* (L/mg) is the Langmuir adsorption equilibrium constant. *Q_m_* is the maximum adsorption capacity of the MAR. *K_F_* is the Freundlich rate constant and *1/n* is an empirical constant related to the adsorption driving force. *K_T_* is the adsorption equilibrium constant in the Temkin equation, where *B_T_ = RT/b*, *R* is the gas constant, *T* is the temperature and *b* is the Temkin constant.

Calculating the thermodynamic parameters of the adsorption process is crucial for understanding its thermodynamic nature. The key parameters are the enthalpy change Δ*H* (KJ/mol), free energy change Δ*G* [KJ/(mol·K)], and entropy change Δ*S* (KJ·mol). These parameters were determined using the following equations [28]:

(11) $$\Delta G=-RT\ln K_{c}$$

(12) $$\ln K_{c}=-\frac{\Delta H}{RT}+\frac{\Delta S}{R}$$

where *R* is the gas constant of 8.314 J/(mol·K), *T* is the thermodynamic temperature, and *K_c_* is the equilibrium constant.

#### 2.8.4. Screening of Desorption Concentrations

Ethanol, known for its low toxicity and high efficacy in eluting active compounds from MAR, was selected as the desorption solvent for this experiment. To identify the optimal ethanol concentration for desorption, a range of concentrations was systematically evaluated, including 10%, 20%, 30%, 40%, 50%, 60%, 70%, 80%, 90%, and 95% (*v*/*v*).

### 2.9. Dynamic Adsorption and Desorption of MAR HPD-600

The concentration of the up-sampling solution is the optimal concentration based on the preliminary explorations in the previous period. A chromatographic column (1.0 cm × 30 cm) with a packing bed volume (BV) of 12 mL was used for the dynamic adsorption experiments. The flow rates were set to 2, 4, and 6 BV/h to assess their effects on the adsorption capacity. Samples were collected in 10-mL tubes, and their triterpenoid contents were measured. The leakage point was identified when the triterpenoid content reached 1/10 of the initial sample concentration, allowing for the determination of the adsorption volume and the optimal flow rate [29]. At the optimal flow rate and sample volume, elution was performed using 70% (*v*/*v*) ethanol at various flow rates, and the triterpenoid content was measured to establish the optimal elution flow rate.

### 2.10. UPLC-Q-TOF-MS Conditions

After the sample solution was filtered through a 0.22 μm membrane filter, the components of the enriched substance were analyzed by UPLC-Q-TOF-MS. The determination conditions were based on the method of Pang et al. with some slight modifications [30]. An ACQUITY UPLC^®^ HSS C18 chromatographic column (2.1 mm × 100 mm, 1.7 μm) was used; the flow rate was 0.35 mL/min, the column temperature was 25 °C, the injection volume was 10 μL, and the mobile phase consisted of acetonitrile (A) and a 0.1% formic acid solution (B). The elution gradient was as follows: 2% A (0~1 min), 2~98% A (1~12 min), and 98~2% A (12~15 min). The analysis was carried out using an electrospray ionization source (ESI) in the negative ion scanning mode. The mass scanning range was *m*/*z* 50~1500, the desolvation gas flow rate was 550 L/h, the nebulizing gas pressure was 50 psi, the ion source temperature was 120 °C, and the desolvation temperature was 450 °C.

### 2.11. Statistical Analysis

All tests in this experiment were performed in triplicate, and basic data analysis was performed using Excel software. The plots were generated using Origin 2022. Significance analyses were performed with SPSS 29.0 software. Different letters in the figures indicate a significant difference (*p* < 0.05), while the same letters indicate a nonsignificant difference (*p* > 0.05).

## 3. Results

### 3.1. Screening of I. hispidus Strains with High Triterpenoids Content

**Figure 1:**
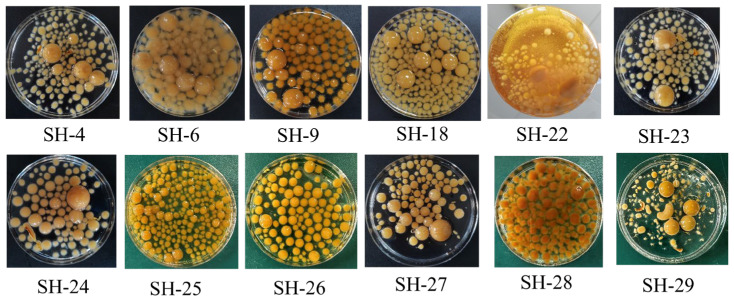
Growth phenotype of the *I. hispidus* strains under the submerged fermentation condition.

**Figure 2:**
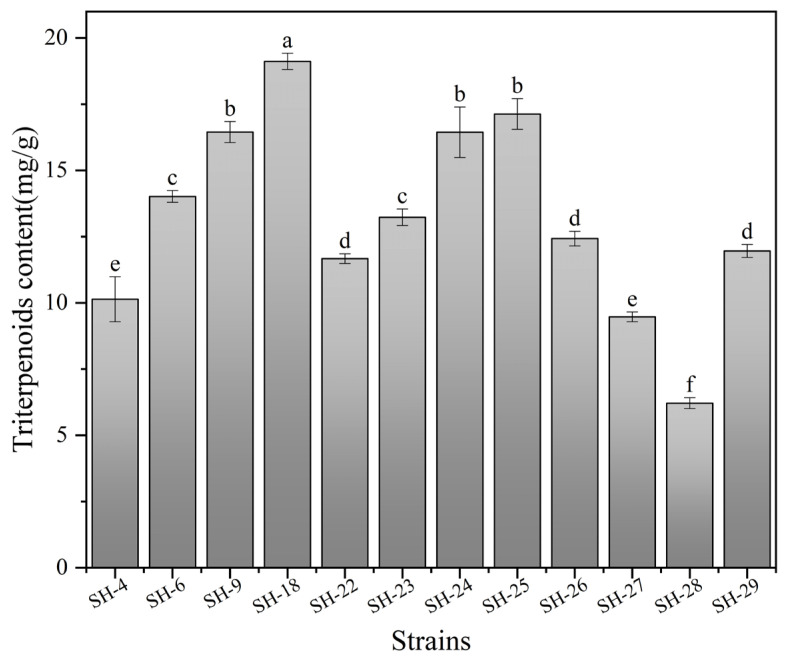
Comparation of the triterpenoid content among 12 strains of *I. hispidus*. Differences in triterpene content among different strains are indicated by different lowercase letters (*p* < 0.05).

Twelve strains of *I. hispidus* isolated from different regions of China were subjected to submerged culture to evaluate their accumulation capacities of triterpenoids. As shown in Figure 1, these strains showed different growth phenotypes under the same culture conditions, although they all belonged to *I. hispidus*. We hypothesized that the variation was mainly due to the effects of evolution under different growth conditions. Compared with several other strains, SH-18 exhibited uniform morphology and stable yield. Simultaneously, the contents of triterpenoids in the mycelium were determined. It was revealed that the triterpenoid content of strain SH-18 was 19.12 ± 0.31 mg/g, which was much higher than that of other strains. Therefore, the SH-18 strain was selected for subsequent study (Figure 2).

### 3.2. Screening of the MAR Fitting for Triterpenoid Enrichment

**Figure 3:**
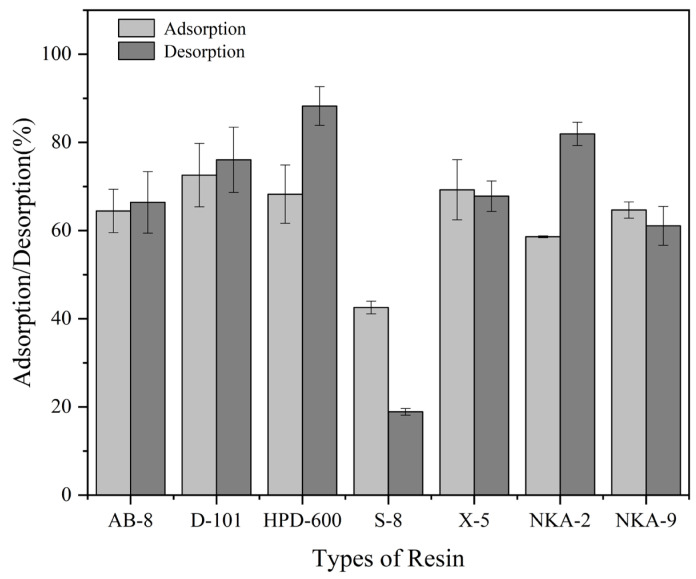
Comparison of the adsorption and desorption rates of triterpenoids across seven MARs.

Both the adsorption and desorption rates are critical indicators for the evaluation of an MAR for substance enrichment. Seven types of MAR with similar particle diameters were selected for triterpenoid enrichment. According to Figure 3, the adsorption rates for the MARs were 61.46% for AB-8, 72.58% for D-101, 68.26% for HPD-600, 42.55% for S-8, 69.25% for X-5, 58.61% for NKA-2, and 64.67% for NKA-9. The desorption rates were 66.40%, 76.06%, 88.24%, 18.91%, 67.82%, 81.93%, and 61.09% for AB-8, D-101, HPD-600, S-8, X-5, NKA-2, and NKA-9, respectively. Among the data, it could be found that the MAR D-101 exhibited the highest adsorption capacity, followed by HPD-600. The specific surface area, average pore diameter, and polarity are three key factors affecting the adsorption and desorption performance of an MAR [31]. MARs with smaller pore sizes and larger specific surface areas have more pores per unit area, which can enhance their ability to adsorb the target substance and ensure better contact with the desorption agent. Both MAR D-101 and HPD-600 have smaller average pore diameters and larger specific surface areas, providing ample adsorption sites for the target substances. Therefore, they showed good adsorption capacity among the seven MARs. In contrast, MAR S-8 and NKA-2 exhibited poorer adsorption of triterpenoids, which may be mainly due to their smaller specific surface areas. Simultaneously, MAR HPD-600 showed the best desorption performance of triterpenoids, indicating that it could effectively desorb adsorbed triterpenoids. Conversely, MAR NKA-2 showed a weaker affinity for triterpenoids, which might be primarily due to its higher desorption rate. Consequently, MAR HPD-600 was the preferred choice for subsequent adsorption kinetics analysis through the above evaluation.

### 3.3. Static Adsorption and Desorption Tests for Selected MAR

Theoretical investigation on the adsorption of a substance by an MAR is important for deeply understanding the underlying mechanisms, including the interactions between the adsorbent and the target compounds, as well as the factors influencing adsorption capacity. Currently, the corresponding study primarily includes analysis of the thermodynamics and kinetics of substance adsorption. Kinetic study can examine the adsorption mechanism and diffusion behavior of an MAR during substance adsorption, which is typically analyzed by pseudo-first-order kinetics, pseudo-second-order kinetics, and the intraparticle diffusion model. Thermodynamic study can investigate the adsorption mode of an MAR on the target substance through fitting adsorption isotherms to models such as the Langmuir model and Freundlich model.

#### 3.3.1. Adsorption Kinetics of MAR HPD-600

**Figure 4:**
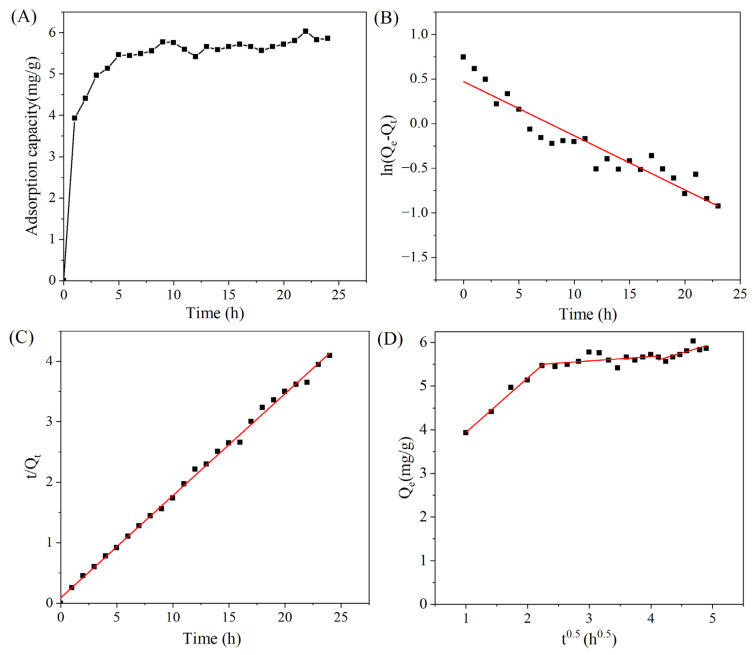
Adsorption kinetic curve (**A**) and linear correlations based on pseudo-first-order (**B**), pseudo-second-order (**C**), and intraparticle diffusion (**D**) equations for the enrichment of total triterpenes using MAR HPD-600 at 30 °C.

The adsorption process is multi-staged and dynamic rather than steady. Typically, the target substance diffuses from the boundary layer of the MAR into its pores and adsorbs onto the active sites on the resin surface. The adsorption rate is influenced by both the adsorption time and solvent used. The adsorption kinetics curve illustrates how the adsorption state changes over time. As shown in Figure 4A, the quantity of triterpenoids adsorbed increased progressively with time. Between 0 and 5 h, the adsorption of triterpenoids onto the MAR HPD-600 increased considerably, reaching 5.13 ± 0.12 mg/g. Beyond the 5-h mark, although the overall adsorption amount continued to increase, the growth rate was considerably slower than that in the initial phase, indicating that the adsorption process was nearing saturation. The character of the process was similar to the report by Li et al. (2024) [23].The surface of the MAR initially had numerous unoccupied sites available for triterpenoids binding. As the adsorption progressed, these sites were occupied, which reduced the number of available binding sites, thus lowering the adsorption efficiency. Therefore, the optimal equilibrium time for triterpenoid adsorption on MAR HPD-600 was 5 h.

Kinetic analysis can predict adsorption outcomes and provide insights into the adsorption behavior and mechanisms of resins. In this study, the adsorption process was evaluated using pseudo-first-order, pseudo-second-order, and intraparticle diffusion models. The pseudo-first-order model is generally used to analyze the initial stage of adsorption, and the pseudo-second-order model is used to assess the entire adsorption process [32]. As shown in Table 2, the coefficient of determination (R^2^ = 0.9976) for the pseudo-second-order model was considerably higher than that for the pseudo-first-order model (R^2^ = 0.8960) and the intraparticle diffusion model (R^2^ = 0.9880, 0.2575, and 0.6167). The fitting curve for the intraparticle diffusion model did not pass through the origin, indicating that intraparticle diffusion was not the sole rate-limiting step. Moreover, the multistage fitting characteristics indicated that the adsorption process involved not only surface diffusion but also mesopore and micropore diffusion (Figure 4D). The pseudo-second-order model provided a better linear correlation, with the theoretically simulated adsorption amount of 5.92 mg/g closely matching the actual value of 5.27 ± 0.13 mg/g. Therefore, the pseudo-second-order model could describe the adsorption mechanism of the triterpenoid enrichment process better. Furthermore, it indicated that the adsorption rate was related to the specific surface area and particle size of the adsorbent.

#### 3.3.2. Adsorption Isotherms of MAR HPD-600

**Figure 5:**
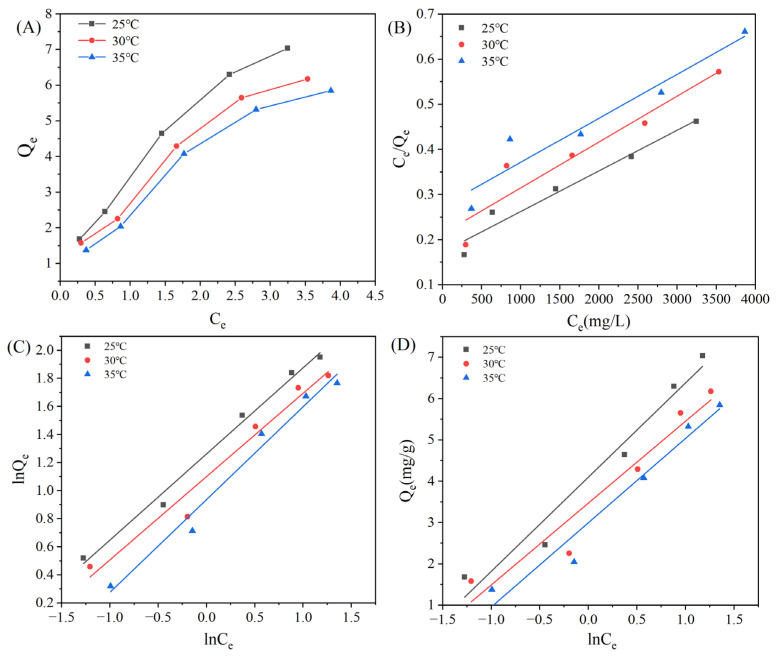
Adsorption isotherms (**A**) and their linear correlations based on the Langmuir (**B**), Freundlich (**C**), and Temkin (**D**) equations for the enrichment of total triterpenoids using MAR HPD-600 at 25 °C, 30 °C, and 35 °C.

To elucidate the adsorption mechanism of *I. hispidus* triterpenoids by MAR HPD-600 at different temperatures, adsorption isotherms were separately plotted at 25 °C, 30 °C and 35 °C. As presented in Figure 5A, the concentration of triterpenoids of *I. hispidus* adsorbed by MAR HPD-600 showed a positive correlation with the increase of the equilibrium concentrations but decreased with the increase of temperature. The result was consistent with the findings reported by Liu et al. (2023) [33]. The result indicated that the adsorption of triterpenoids of *I*. *hispidus* onto MAR HPD-600 was an exothermic process, and the higher the temperature, the lower the adsorption efficiency. Moreover, it revealed that, at a constant temperature, the equilibrium concentration was positively correlated with the content of triterpenoids adsorbed by MAR HPD-600, whereas it was negatively correlated with the adsorption rate.

Furthermore, the linear correlations of triterpenoid adsorption by MAR HPD-600 at 25 °C, 30 °C and 35 °C were analyzed using the Langmuir, Freundlich and Temkin equations. The Langmuir model assumes that adsorption occurs on a homogeneous surface in a monomolecular layer with no interaction between adsorbent molecules [34]. The Freundlich model is an empirical approach used to describe nonideal adsorption processes, which characterizes adsorption as occurring in multiple molecular layers on a nonuniform surface [35]. Temkin equations describe the relationship between heat of adsorption and coverage [36]. From the linear fitting results of the Langmuir model, it could be found that the adsorption decreased as the temperature increased, indicating that exothermic heat enhanced the adsorption process (Figure 5B). In view of the parameters of the three models (Table 3), the linear correlation coefficient R^2^ for the Freundlich model was considerably higher than that of the Langmuir and Temkin models, which meant that this model could more accurately fit the adsorption process of MAR HPD-600 on *I*. *hispidus* triterpenoids than the Langmuir and Temkin models. In this sense, this indicated that adsorption involved multiple molecular layers on a nonuniform surface rather than occurring at a single specific site. Simultaneously, the constant 1/n related to the ease of adsorption; when 0.1 < 1/n < 1, adsorption was relatively easy, while 1/n > 1 revealed more difficult adsorption [35]. The results showed that 1/n was approximately 0.5 at all experimental temperatures, showing that the triterpenoids were readily adsorbed onto the MAR HPD-600. Among these temperatures, 25 °C was found to be the most favorable for the adsorption of triterpenoids of *I*. *hispidus*. Additionally, the decrease in *K_T_* with increasing temperature further confirmed that the adsorption process was exothermic.

**Figure 6:**
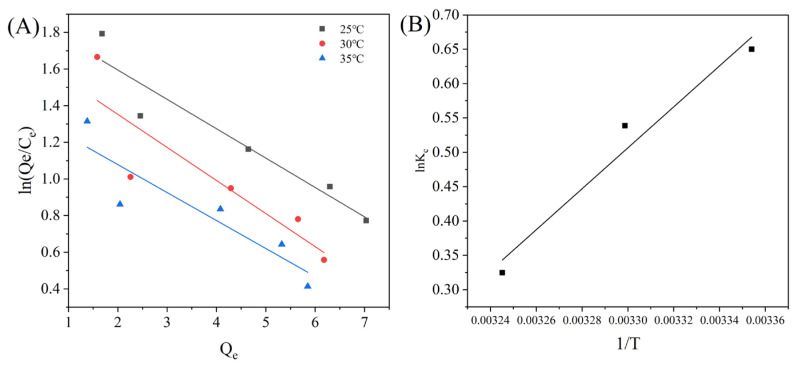
Linear relationship between Q_e_ and ln (Q_e_/C_e_) (**A**), 1/T, and lnK_c_ (**B**).

Figure 6B shows that both Kc values and Δ*G* decreased as the temperature increased, revealing that adsorption reactions were favorable at lower temperatures. Considering that Δ*G* < 0 and Δ*H* < 0 (Table 4), it was concluded that the adsorption process was a spontaneous exothermic reaction, whereas Δ*S* < 0 indicated that the adsorption of the triterpenoids on MAR HPD-600 was less random. Additionally, the range of Δ*G* was between −20 and 0 kJ/mol, revealing that the adsorption was primarily physical process rather than chemical process. This meant that minimal chemical bonding occurred between the triterpenoids and MAR HPD-600, which facilitated the reclamation and reuse of the MAR [37].

#### 3.3.3. Screening of Desorbent Concentrations of MAR HPD-600

**Figure 7:**
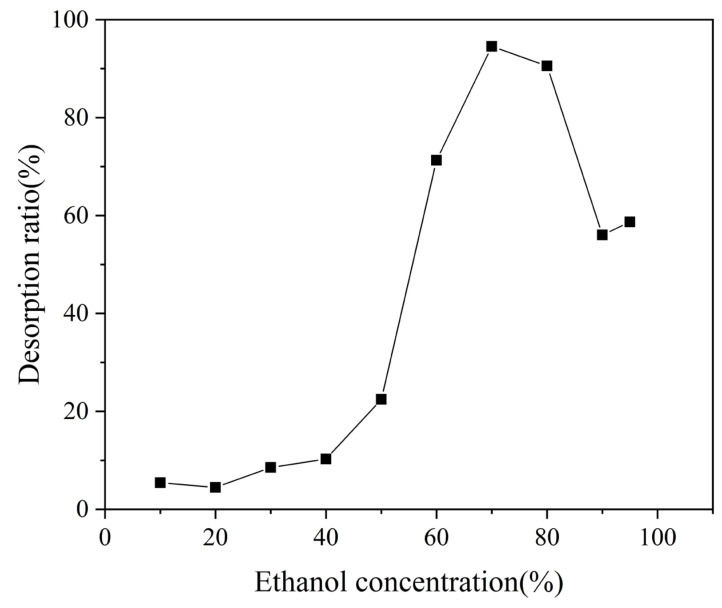
Desorption of triterpenoids from SH-18 using various ethanol concentrations.

Ethanol is a safe, cost-effective, and mild eluent that minimally affects the activity of substances. Additionally, ethanol can be easily recovered and reused through rotary evaporation, making it highly suitable for industrial application. To determine the optimal desorption effect, ethanol concentrations ranging from 10% to 95% (*v*/*v*) were used to desorb the triterpenoids of *I*. *hispidus* on MAR HPD-600, and the desorption efficiencies at different ethanol concentrations were compared. As shown in Figure 7, the desorption efficiency of MAR HPD-600 initially increased with increasing ethanol concentration but then decreased. The desorption rate gradually increased from 10% to 50% ethanol concentration, but it was not more than 20%. These lower efficiencies were likely attributed to the low polarity of triterpenoids, because low-polarity solvents hinder the desorption of target substances from MAR. When the ethanol concentration reached 60%, the desorption rate increased considerably, peaking at 70% and reaching a maximum value of 94.55 ± 0.21%. This revealed that high ethanol concentrations could effectively disrupt the interaction between the triterpenoids of *I. hispidus* and MAR HPD-600, enhancing the desorption process. However, excessively high ethanol concentrations may weaken this effect, leading to incomplete desorption of the triterpenoids. One possible explanation is that when the ethanol concentration was very high, the enhanced intermolecular interactions between ethanol molecules hindered the exchange of triterpenoids adsorbed onto the MAR HPD-600 with ethanol molecules, thereby reducing the desorption efficiency. Therefore, 70% ethanol was selected as the optimal desorption solvent.

### 3.4. Dynamic Adsorption and Desorption

**Figure 8:**
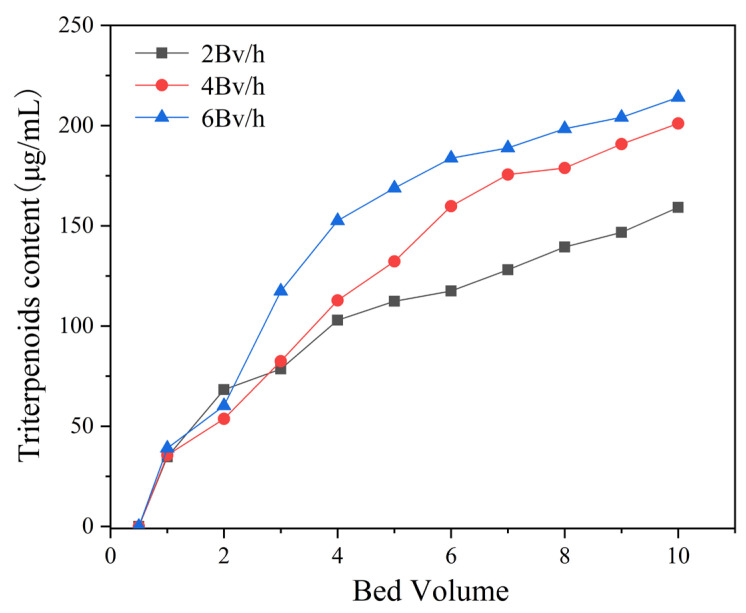
Leakage curves of triterpenoids on MAR HPD-600.

To determine the optimal up-sampling volume and flow rate, dynamic leakage curves of the MAR HPD-600 were established at flow rates of 2, 4, and 6 BV/h. The concentration of *I. hispidus* triterpenoids in the effluent gradually increased as the sample injection continued. The leakage point was identified as being when the effluent concentration reached 10% of the initial sample concentration. At this point, the adsorption ability of the triterpenoids of MAR HPD-600 decreased, leading to a reduction in effectiveness and loss of triterpenoids [38]. The initial triterpenoid content of the sample solution was measured as 1.34 mg/mL. The leakage point was determined when the triterpenoid content in the effluent reached 0.13 mg/mL, which established the optimal sample volume. As shown in Figure 8, the leakage points corresponding to flow rates of 2, 4, and 6 BV/h were 7.5, 5 and 3.5 BV, respectively. The result showed that a lower flow rate promoted better diffusion and adsorption of triterpenoids in the MAR HPD-600, allowing the binding sites of MAR to fully engage with the adsorbent. Conversely, although higher flow rates reduced the dynamic adsorption time, the binding efficiency was compromised. Hence, 4 BV/h was selected as the optimal up-sampling flow rate with an up-sampling volume of 5 BV.

**Figure 9:**
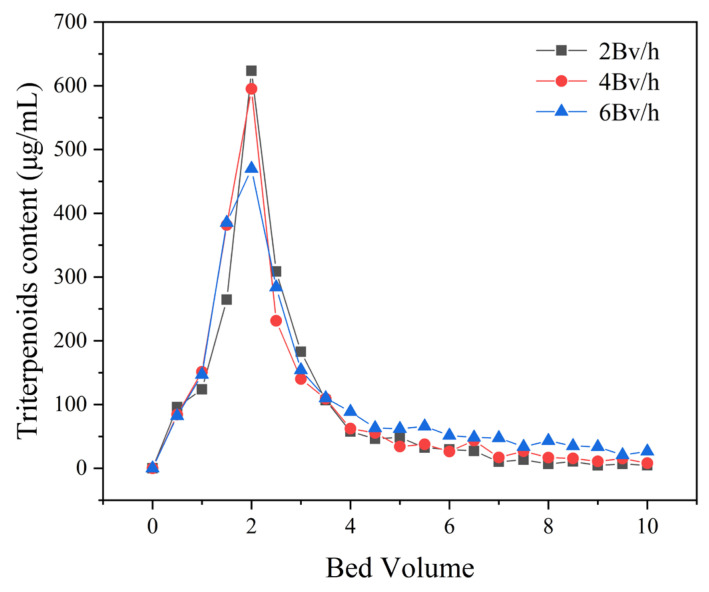
Desorption curves of triterpenoids on MAR HPD-600.

With the aim of obtaining the optimal desorption flow rate and volume, dynamic desorption curves were plotted at flow rates of 2, 4 and 6 BV/h (Figure 9). The triterpenoid content initially increased and then decreased during desorption, with the highest content observed at 2 BV for all flow rates. The optimal eluent volume was defined as the point at which the total triterpenoid content in the eluent reached 5% of the sample solution concentration. For flow rates of 2, 4 and 6 BV/h, the corresponding eluent volumes were 4, 4.5 and 5.5 BV, respectively. It suggested that a lower flow rate allows for more complete dissolution of triterpenoids in the eluent, thus reducing the overall volume of the eluent. However, excessively low flow rates reduce desorption efficiency, which is not ideal for large-scale production. The overall trend at 4 BV/h was similar to that at 2 BV/h; therefore, by balancing the triterpenoid desorption amount with the production efficiency, a flow rate of 4 BV/h was selected, with a desorption volume of 4.5 BV. At this flow rate, the desorption solution contained 129.28 mg/g of triterpenoids compared with the initial content of 26.72 mg/g, resulting in a yield of 75.48%, which is the highest yield reported to date. Wang et al. [39] used a combination of the ionic liquid [BMIM]Br-MeOH to extract all of the triterpenoids from *Inonotus obliquus*. After optimizing the extraction conditions using the response surface methodology, the total triterpenoid content was 16.78 mg/g [39]. There is a gap in the extraction effect when compared with that of this study. Reusability is an important index for the industrial application of MAR. Liu et al. 2023 investigated the reusability of MAR LSA-21 and showed that it could still achieve 94% yield of glabridin after four instances of reuse, which was only 3.8% lower compared with the first use [33]. MAR HPD-600 also showed good reusability for the enrichment of *I. hispidus* triterpenoids after seven instances of reuse. Specifically, the yield decreased by only 2.4%. Through aperture matching and surface adsorption, MAR can specifically adsorb the triterpenoids of *I*. *hispidus* with hydrophobic properties. Compared with the traditional organic solvent extraction method, this method reduces the use of organic solvents such as acetone and petroleum ether, decreases the risk of environmental pollution, and is in line with the concept of green chemistry. Moreover, MARs exhibit stable performance and high enrichment efficiency, and can be reused multiple times, which is crucial for reducing the production costs of continuous industrial operations in food production. Research studies have demonstrated that the use of MARs for the separation and enrichment of plant-derived bioactive compounds facilitates scale-up production [40,41]. For functional foods, MAR can enhance their performance, and the content of triterpenoids after purification can be controlled, which facilitates the formulation of standardized recipes and ensures the consistency of efficacy and compliance of functional foods. Based on this, we believe that the application of MAR for the separation and enrichment of *I. hispidus* triterpenoids also holds significant industrial potential.

### 3.5. Compositional Analysis of the Purified Substances

**Figure 10:**
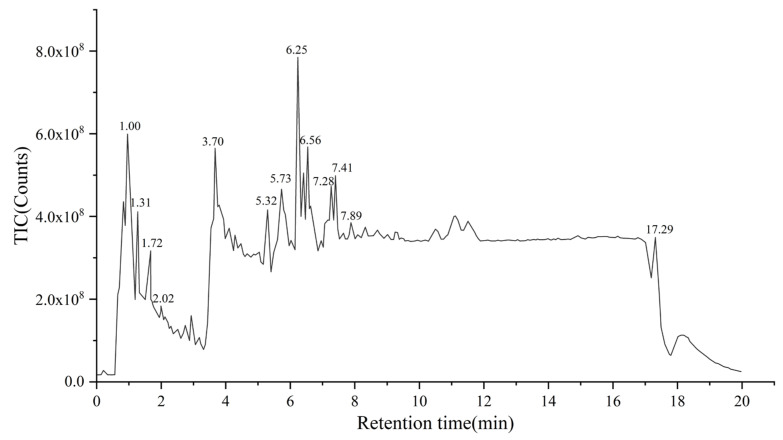
Chromatogram of TIC analyzed by UPLC-Q-TOF-MS.

Qualitative analysis of the enrichment was carried out using UPLC-Q-TOF-MS. The total ion flow diagram obtained revealed that the purified product consisted of a variety of compounds (Figure 10). Using Waters UNIFI platform for automatic compound identification, a total of four triterpenoids were tentatively identified in the enrichment, and the corresponding information of substances was provided in Table 5. These compounds, like Olibanumols J, exhibited remarkable hypoglycemic activity. [42]. Moreover, trace amounts of non-triterpenoid compounds, including sesquiterpenoids, diterpenoids, and alkaloids, were also present. These compounds also possessed potential biological activities. This characteristic was in line with the development trend of a ‘multi-target synergistic effect’ in the development of natural bioactive products. This conception breaks through the traditional pharmaceutical approach of ‘one drug, one target, one disease’, addresses limitations in disease treatment such as drug resistance and side effects of combined drugs, and enhances the efficacy of disease treatment. In the context of functional foods, the multi-target synergistic effect strategy can not only comprehensively regulate physiological functions, resulting in overall health benefits greater than the sum of individual components’ effects, but also reduce the potential side effects associated with the use of a single high-dose component. This strategy simultaneously enables the intervention and prevention of multiple diseases, bringing more benefits to consumers [43,44,45].

## 4. Conclusions

An effective method for extracting and purifying triterpenoids from *I. hispidus* using MAR was established in this study. MAR HPD-600 demonstrated the highest performance, with adsorption and desorption rates of 68.26% and 88.24%, respectively. The pseudo-second-order kinetic model exhibited a significantly higher coefficient of determination compared to the pseudo-first-order and intraparticle diffusion models. The Freundlich model provided the best fit for the adsorption isotherms, suggesting that the adsorption of *I. hispidus* triterpenoids by MAR HPD-600 is a spontaneous exothermic process, characterized by regular multimolecular layer adsorption.

Upon evaluating both the yield and production efficiency of triterpenoids, the up-sampling volume was set at 5 BV according to the leakage point. Through comparing the triterpenoid content in the desorption solutions, the volume of the desorption solution was found to be 4.5 BV, with a 70% (*v*/*v*) ethanol solution used. Under these optimal conditions, the content of triterpenoids from *I. hispidus* increased from 26.72 to 129.28 mg/g, representing a 3.8-fold increase, with a high yield of 75.48%.

In conclusion, this study presents a method that is characterized by simplicity, cost-effectiveness, high recovery efficiency and standardization, offering valuable theoretical guidance for the industrial application of MAR in the enrichment of *I. hispidus* triterpenoids. Compared with silica gel column chromatography, the method developed in this research exhibits distinct merits in terms of safety, cost-efficiency, and compatibility with large-scale industrial production within the functional food field. However, the adsorption selectivity of MAR for triterpenoid compounds is limited, and it may simultaneously adsorb impurities such as polysaccharides and pigments, resulting in an increase in the difficulty of subsequent purification. To obtain a product with higher purity, other separation methods can be combined for further purification. In the event that highly pure individual triterpenoids are a requisite, or when considering applications within the realm of pharmaceutical development, additional chromatographic purification procedures are indispensable.

Triterpenoids such as ginsenosides and betulin have been proven to have the effects of prolonging lifespan and delaying aging, demonstrating that triterpenoids can serve as potential geroprotectors [46,47,48,49]. Meanwhile, triterpenoids have broad application prospects in enhancing food functionality, food preservation and flavor regulation [50]. Therefore, relevant health foods can be developed based on the activities of triterpenoids from *I. hispidus*. The triterpenoids in the fruiting bodies of *I. hispidus* can inhibit the production of NO in LPS-induced BV-2 microglial cells. They can be added to some functional beverages targeted at the elderly or high-risk groups for neurological diseases, such as brain-tonifying oral liquids [51]. The triterpenoids in *I. obliquus* can significantly inhibit the activity of α-glucosidase and can be developed as a metabolic regulator for related anti-diabetic foods and meal replacement powders [52]. In the future, the commercial potential in segmented markets such as anti-aging and metabolic health can be further expanded through precise targeted delivery systems and clinical efficacy verification.

## Figures and Tables

**Table 1:** Performance characteristics of the MARs.

MAR	Surface Area (m^2^/g)	Average Pore Diameter (nm)	Particle Diameter (mm)	Polarity
AB-8	480–520	13–14	0.3–1.25	Weak polar
D-101	500–550	9–11	0.3–1.25	Non-polar
HPD-600	550–600	8–9	0.3–1.25	Weak polar
S-8	100–120	28–30	0.3–1.25	Polar
X-5	500–600	29–30	0.3–1.25	Weak polar
NKA-2	160–200	14.5–15.5	0.3–1.25	Polar
NKA-9	250–290	15.5–16.5	0.3–1.25	Polar

**Table 2:** Kinetic model fitting equations and parameters for the adsorption of the total triterpenes on MAR HPD-600 resin.

Models	Equations	Parameters
Pseudo-first-order	$\ln\left(Q_{e}-Q_{t}\right)=-0.061t+0.472$	*K*_1_ = 0.0265 h^−1^
		R^2^ = 0.8960
		*Q_e_* = 2.96 mg/g
Pseudo-second-order	$t/Q_{t}=0.169t+0.090$	*K*_2_ = 0.0026 h^−1^
		R^2^ = 0.9976
		*Q_e_* = 5.92 mg/g
Intraparticle diffusion	$Q_{t}=1.25t^{0.5}+2.69$	R^2^ = 0.9880
	$Q_{t}=0.10t^{0.5}+5.28$	R^2^ = 0.2575
	$Q_{t}=0.42t^{0.5}+3.85$	R^2^ = 0.6167

**Table 3:** Kinetic model fitting equations and parameters for the adsorption of total triterpenoids on MAR HPD-600 resin at 25 °C, 30 °C, and 35 °C.

Models	T (°C)	Equations	Parameters		
			*Q_m_* (mg/g)	*K_L_* (L/mg)	R^2^
Langmuir	25	CeQe=0.09008Ce+0.1722	11.10	0.5232	0.9602
	30	CeQe=0.01017Ce+0.2129	98.33	0.0478	0.90031
	35	CeQe=0.09745Ce+0.2739	10.26	0.3558	0.92091
			n	*K_F_* [(mg/g) (mL/mg)1/n]	R^2^
Freundlich	25	$\ln Q_{e}=0.61355\ln C_{e}+1.26159$	1.6299	3.5310	0.99081
	30	$\ln Q_{e}=0.59324\ln C_{e}+1.1003$	1.6857	3.0051	0.96987
	35	$\ln Q_{e}=0.66099\ln C_{e}+0.93652$	1.5129	2.5511	0.97918
			*B_T_*	*K_T_* (mL/mg)	R^2^
Temkin	25	$Q_{e}=2.2812\ln C_{e}+4.0980$	2.2812	6.0280	0.96245
	30	$Q_{e}=1.9822\ln C_{e}+3.4701$	1.9822	5.7582	0.93338
	35	$Q_{e}=2.0408\ln C_{e}+2.9952$	2.0408	4.3391	0.95733

**Table 4:** Thermodynamic parameters of total triterpenes adsorbed on MAR HPD-600 at 25 °C, 30 °C, and 35 °C.

Δ*G* (KJ/mol)			Δ*H* (KJ/mol)	Δ*S* (J/mol)
25 °C	30 °C	35 °C	−0.36	−1.12
−4.75	−4.32	−3.54		

**Table 5:** The identification results of triterpenoids in MAR HPD-600 enriched material.

Number	t_R_ (min)	Observed (*m*/*z*)	Formula	Error (mDa)	Name
1	5.6	544.381	C_32_H_52_O_5_	−2.7	16β-Methoxyalisol B 23-acetate
2	5.72	461.3996	C_30_H_52_O_3_	0.6	Olibanumols J
3	5.87	503.409	C_24_H_38_O_4_	−0.9	(20S,24R)-3β-O-Acetyl-20,25-epoxydammarane-24-ol
4	6.51	503.4084	C_24_H_38_O_4_	−1.1	Ocotillol acetate

## Data Availability

The original contributions presented in the study are included in the article, further inquiries can be directed to the corresponding author.
